# Critical Role of TGF-β and IL-2 Receptor Signaling in Foxp3 Induction by an Inhibitor of DNA Methylation

**DOI:** 10.3389/fimmu.2018.00125

**Published:** 2018-02-02

**Authors:** Kristin Freudenberg, Nadja Lindner, Sebastian Dohnke, Annette I. Garbe, Sonja Schallenberg, Karsten Kretschmer

**Affiliations:** ^1^Molecular and Cellular Immunology/Immune Regulation, DFG-Center for Regenerative Therapies Dresden (CRTD), Center for Molecular and Cellular Bioengineering (CMCB), Technische Universität Dresden, Dresden, Germany; ^2^Osteoimmunology, DFG-Center for Regenerative Therapies Dresden (CRTD), Center for Molecular and Cellular Bioengineering (CMCB), Technische Universität Dresden, Dresden, Germany; ^3^Paul Langerhans Institute Dresden (PLID) of the Helmholtz Zentrum München at the University Hospital and Medical Faculty Carl Gustav Carus of TU Dresden, Dresden, Germany; ^4^German Center for Diabetes Research (DZD e.V.), Neuherberg, Germany

**Keywords:** epigenetic regulation, iTreg, Foxp3, DNA methylation, CNS2

## Abstract

Under physiological conditions, CD4^+^ regulatory T (Treg) cells expressing the transcription factor Foxp3 are generated in the thymus [thymus-derived Foxp3^+^ Treg (tTregs) cells] and extrathymically at peripheral sites [peripherally induced Foxp3^+^ Treg (pTreg) cell], and both developmental subsets play non-redundant roles in maintaining self-tolerance throughout life. In addition, a variety of experimental *in vitro* and *in vivo* modalities can extrathymically elicit a Foxp3^+^ Treg cell phenotype in peripheral CD4^+^Foxp3^−^ T cells, which has attracted much interest as an approach toward cell-based therapy in clinical settings of undesired immune responses. A particularly notable example is the *in vitro* induction of Foxp3 expression and Treg cell activity (iTreg cells) in initially naive CD4^+^Foxp3^−^ T cells through T cell receptor (TCR) and IL-2R ligation, in the presence of exogenous TGF-β. Clinical application of Foxp3^+^ iTreg cells has been hampered by the fact that TGF-β-driven Foxp3 induction is not sufficient to fully recapitulate the epigenetic and transcriptional signature of *in vivo* induced Foxp3^+^ tTreg and pTreg cells, which includes the failure to imprint iTreg cells with stable Foxp3 expression. This hurdle can be potentially overcome by pharmacological interference with DNA methyltransferase activity and CpG methylation [e.g., by the cytosine nucleoside analog 5-aza-2′-deoxycytidine (5-aza-dC)] to stabilize TGF-β-induced Foxp3 expression and to promote a Foxp3^+^ iTreg cell phenotype even in the absence of added TGF-β. However, the molecular mechanisms of 5-aza-dC-mediated Foxp3^+^ iTreg cell generation have remained incompletely understood. Here, we show that in the absence of exogenously added TGF-β and IL-2, efficient 5-aza-dC-mediated Foxp3^+^ iTreg cell generation from TCR-stimulated CD4^+^Foxp3^−^ T cells is critically dependent on TGF-βR and IL-2R signaling and that this process is driven by TGF-β and IL-2, which could either be FCS derived or produced by T cells on TCR stimulation. Overall, these findings contribute to our understanding of the molecular mechanisms underlying the process of Foxp3 induction and may provide a rational basis for generating phenotypically and functionally stable iTreg cells.

## Introduction

Peripherally induced Foxp3^+^ Treg (pTreg) cells, which are generated from precommitted CD4^+^Foxp3^−^CD25^+^ pTreg cell precursors (1, 2), comprise 20–40% of the mature Treg cell pool in steady-state mice (3) and act in concert with thymus-derived Foxp3^+^ Treg cells (tTregs) to enforce immune tolerance (3–6). In addition to such naturally occurring pTreg cells that continuously develop in peripheral lymphoid tissues of nonmanipulated mice, Foxp3^+^ pTreg cells can be artificially generated *in vivo* from post-thymic, initially naive CD4^+^Foxp3^−^ T cells in experimental settings of lymphopenia-driven proliferation (7, 8) and subimmunogenic antigen administration (9, 10).

Early studies using CD25 as a surrogate Treg cell marker provided first evidence that CD4^+^CD25^−^ T cells (11, 12) can acquire a Treg cell phenotype *in vitro* [termed iTreg cells (13)] upon T cell receptor (TCR) stimulation in the presence of added TGF-β. After anti-Foxp3 mAbs and Foxp3-fluorochrome reporter mice became commonly available, numerous reports have extended the concept of TGF-β-/TCR-mediated Foxp3^+^ induction to truly naive CD4^+^Foxp3^−^ T cells by rigorously excluding pre-formed Foxp3^+^ Treg cells. These studies established that the process of TGF-β-/TCR-mediated Foxp3^+^ iTreg cell generation is strictly dependent on IL-2R signaling and IL-2, which could either be exogenously added or produced by TCR-stimulated CD4^+^ T cells (14). Enhanced co-stimulation and inflammatory cytokines antagonize TGF-β-mediated Foxp3 induction, whereas retinoic acid augments this process through direct and indirect mechanisms (15), including interference with inhibitory cytokine signaling (16) and enhanced TGF-β-driven SMAD3 phosphorylation in developing iTreg cells (17), as well as inhibition of inflammatory cytokine secretion by bystander cells (18). Since then, TGF-β-mediated iTreg cell generation has been widely used as an experimental *in vitro* approach that recapitulates some aspects of Foxp3^+^ Treg cell development *in vivo*. However, TGF-β-mediated Foxp3^+^ iTreg cell generation *in vitro* fails to recapitulate the epigenetic (19–23) and transcriptional (24, 25) signature of *in vivo* generated Foxp3^+^ Treg cells, which is reflected by variable suppressor activity (26) and unstable Foxp3 expression (19, 20, 27), precluding the clinical use of TGF-β-induced Foxp3^+^ iTreg cells.

While the rather weak transactivation activity of the murine basal *Foxp3* promoter may help prevent promiscuous Foxp3 expression, extrathymic induction and maintenance of Foxp3 expression is facilitated by two cis-acting, conserved non-coding regions (CNS) within the *Foxp3* gene locus that exhibit enhancer activity. CNS1 contains binding sites for transcription factors activated downstream of three major signaling pathways implicated in this process (TCR: NFAT; TGF-βR: Smad3; retinoid acid: RAR/RXR) (28), and Smad binding to CNS1 mediates TGF-β responsiveness of developing iTreg and pTreg cells (5, 29). When ≤0.5 ng/ml TGF-β is added, initially naive CD4^+^Foxp3^−^ T cells selectively lacking the TGF-β-Smad response element (Foxp3-CNS1^mut^) (29) or the entire CNS1 (Foxp3Δ^CNS1^) (5, 30) exhibit substantially impaired Foxp3^+^ iTreg cell generation; increasing TGF-β concentrations partially compensate for this defect (5, 29), with ≥1 ng/ml resulting in comparable proportions of wild-type and Foxp3-CNS1^mut^ Foxp3^+^ iTreg cells (29). In contrast to the essential role of CNS1 in extrathymic (but not intrathymic) Foxp3 induction, CNS2 (also known as Treg-specific demethylated region) contains CpG-rich islands whose efficient demethylation promotes binding of transcription factors (e.g., NFAT, STAT5, Runx1/Cbfβ, CREB, Foxp3 itself), thereby stabilizing upregulated Foxp3 expression. CNS2 becomes intrathymically hypomethylated during early tTreg cell development (31), enabling stable Foxp3 expression in dividing Treg cells outside the thymus, in particular in the presence of inflammatory cytokines or limited IL-2 (32, 33). Strikingly, pTreg cells generated *in vivo* by DEC-205^+^ DC targeting (34) exhibit efficient CNS2 demethylation and overwhelmingly stable Foxp3 expression under inflammatory conditions *in vitro* (20) and *in vivo* (9), whereas initially Foxp3^+^ iTreg cells possess a methylated (or partially demethylated) CNS2 and rapidly lose Foxp3 expression upon TCR restimulation in the absence of added TGF-β (19, 20). CNS2 may further promote Foxp3 expression by NFAT-dependent interaction with the Foxp3 promoter, allowing CNS2-bound STAT5 to access the *Foxp3* promoter (32). IL-2 has also been implicated in stabilizing CNS2 hypomethylation and Foxp3 expression by modulating the recruitment of methylating enzymes (35). The important role of IL-2R signaling in stabilizing Foxp3 expression can potentially be explained by high CD25 expression levels correlating with Foxp3^+^ Treg cell stability (36). Similarly, and besides initiating extrathymic Foxp3 induction through CNS1-Smad binding, TGF-βR signaling has been linked to epigenetic stabilization of CNS2 hypomethylation and induced Foxp3 expression through the suppression of DNA methyltransferases 1 (Dnmt1) expression (37).

While TCR stimulation alone results in rapid Foxp3 downregulation, the Foxp3^+^ phenotype of iTreg cells with strong CNS2 methylation can be preserved by high-dose IL-2 (38) or by continued supply of TGF-β and TCR stimulation (19, 20). Pharmacological inhibition of Dnmt activity through 5-azacytidine (5-aza-C) or its deoxyribose analog 5-Aza-2′-deoxycytidine (5-aza-dC) (39) enhances TGF-β-mediated Foxp3^+^ iTreg cell generation (20, 21) and promotes efficient CNS2 demethylation in Foxp3^+^ but not in neighboring Foxp3^−^ cells (20). Consequently, the majority of Foxp3^+^ iTreg cells generated by TGF-β in the presence of 5-aza-dC maintain Foxp3 expression in IL-2-supplemented TCR re-stimulation cultures (20). Notably, in the presence of IL-2 but in the absence of added TGF-β, interference with CpG methylation by genetic *Dnmt1* ablation (40) or pharmacological inhibition (20, 21, 41, 42) is sufficient to induce Foxp3 expression in TCR-stimulated CD4^+^Foxp3^−^ T cells. Such TCR-/5-aza-dC-induced Foxp3^+^ iTreg cells exhibited markedly enhanced CNS2 demethylation and stable Foxp3^+^ expression upon TCR/IL-2R restimulation (20).

A causal relationship between 5-aza-dC-mediated *Foxp3*-CNS2 demethylation and stabilization of induced Foxp3 expression has been firmly established (19–21, 32, 33), but it has remained unclear whether early events during 5-aza-dC-mediated Foxp3^+^ iTreg cell generation are also mediated through direct mechanisms (i.e., CpG demethylation of the *Foxp3* gene) or by indirectly regulating signaling pathways that then promote Foxp3 induction. On the basis of the observation that 5-aza-dC enhances surface expression of the IL-2Rα subunit CD25 and synergizes with TGF-β in promoting Foxp3^+^ iTreg cell generation, we hypothesized that 5-aza-dC may facilitate Foxp3 induction through TGF-βR and IL-2R signaling. Here, we show that TGF-βR and IL-2R signaling is indispensable for efficient 5-aza-dC-mediated Foxp3^+^ iTreg cell generation. Our results suggest that, in the absence of added cytokines, 5-aza-dC sensitizes converting CD4^+^Foxp3^−^ T cells to undergo TGF-βR and IL-2R signaling-dependent Foxp3 upregulation driven by low amounts of FCS-/T cell-derived TGF-β and IL-2.

## Materials and Methods

### Mice

Mice with transgenic expression of GFP as a fusion protein with Foxp3 (Foxp3^GFP^) (43) and Foxp3^GFP^ mice that additionally expressed a dominant-negative TGF-βRII (dnTGF-βRII) (44) in CD4^+^ and CD8^+^ T cells (Foxp3^GFP^ × dnTGF-β-RII) were on the C57BL/6 CD45.1 background. All mice were housed and bred at the Animal Facility of the CRTD under specific pathogen-free conditions. All animal studies were performed in strict accordance with German Animal Welfare legislation. All protocols were approved by the Institutional Animal Welfare Officer (Tierschutzbeauftragter), and necessary licenses were obtained from the regional license granting body (Landesdirektion Dresden, Germany).

### Flow Cytometry and Cell Sorting

Single-cell suspensions of the spleen, mLN, and a pool of scLN (*Lnn. mandibularis, Lnn. cervicales superficiales, Lnn. axillares et cubiti, Lnn. inguinales superficiales*, and *Lnn. subiliaci*) were prepared using 70-µm cell strainers (BD). mAbs to CD4 (RM4-5, GK1.5), CD8 (53–6.7), CD25 (PC61, 7D4), CD62L (MEL-14), pSTAT5 [Clone 47 recognizing phosphorylated Y694 of Stat5a (Stat5(pY694))] and Pacific Blue- and PE-conjugated streptavidin were obtained from eBioscience or BD. Before FACS, for some experiments, CD4^+^ or CD8^+^ cells were enriched from single-cell suspensions using biotinylated mAbs directed against CD4 or CD8, respectively, streptavidin-conjugated microbeads and the AutoMACS magnetic cell separation system (Miltenyi Biotec). Samples were analyzed on an LSRII or sorted using a FACS Aria II or FACS Aria III (BD). Data were analyzed using FlowJo software (Tree Star, Inc.).

### T Cell Culture

**Figure 1:**
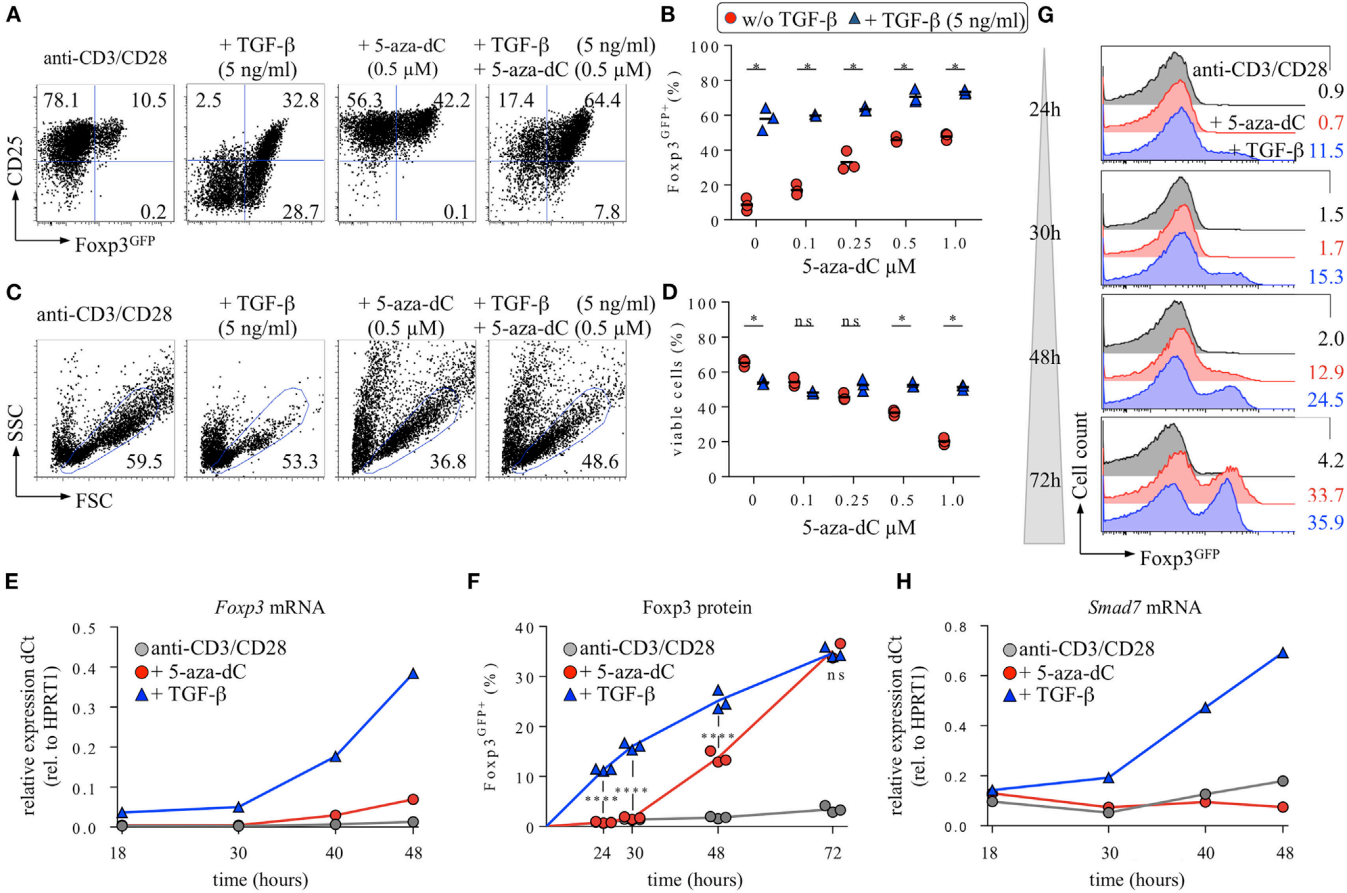
Interplay of 5-aza-2′-deoxycytidine (5-aza-dC) with IL-2R/TGF-βR signaling during Foxp3^+^ iTreg cell generation. CD4^+^Foxp3^GFP−^ T cells with a naive phenotype (CD25^−^CD62L^high^) were FACS-isolated to high purity (99.7%) from pooled lymph nodes and spleen of adult Foxp3^GFP^ mice and subjected to T cell receptor (TCR) stimulation *in vitro* (anti-CD3/CD28-coated beads), either alone or with TGF-β and/or 5-aza-dC, as indicated. Note that all cultures were performed in the absence of exogenous IL-2. At indicated time points, cultures were analyzed for Foxp3^GFP^ and CD25 expression among gated CD4^+^ T cells. **(A–D)** 5-aza-dC synergizes with TGF-β in promoting Foxp3^+^CD25^+^ iTreg cell generation. As indicated, TCR stimulation cultures were either left untreated or supplemented with TGF-β (5 ng/ml) and/or titrating amounts of 5-aza-dC (0.1, 0.25, 0.5, or 1.0 µM). **(A)** Representative flow cytometry of Foxp3^GFP^/CD25 expression among gated CD4^+^ T cells, and **(B)** composite percentages of CD4^+^Foxp3^GFP+^ iTreg cells at day 3 of cultures. **(C)** Corresponding representative flow cytometry of cell viability (FSC/SSC), and **(D)** composite percentages of viable cells within the indicated FSC/SSC gate. The level of significance was determined by multiple *t*-tests (unpaired). *P* ≤ 0.05 (*). **(E–H)** Naive CD4^+^Foxp3^GFP−^ T cell stimulation cultures were either left untreated (gray circles) or supplemented with 0.5 ng/ml TGF-β (blue triangles) or 0.5 µM 5-aza-dC (red circles) and subjected to further analysis at indicated time points. **(E)** Kinetics of *Foxp3* mRNA expression. **(F,G)** Kinetics of Foxp3 protein expression. **(F)** Composite percentages and **(G)** representative histograms of Foxp3^GFP^ expression among gated CD4^+^ T cells at indicated time points and culture conditions. **(H)** Kinetics of *Smad7* mRNA expression. Gene expression **(E,H)** was determined by real-time RT-PCR employing FACS-purified populations of total viable cells (i.e., irrespective of their Foxp3^GFP^ expression status). HPRT1 was used for normalization. Where indicated, the level of significance was determined by two-way ANOVA with Tukey’s multiple comparison test: ****P ≤ 0.0001 and *P* > 0.05 (ns). Numbers **(A,C,G)** show the percentage of gated cells within the respective quadrant or gate. Symbols and horizontal lines **(B,D)** indicate triplicate wells and mean values, respectively. Data are representative of at least two independent experiments.

All cultures were performed with FACS-purified populations of naive T cells (either CD4^+^ or CD8^+^, as indicated) with a naive Foxp3^GFP−^CD25^−^CD62L^high^ phenotype and analyzed at day 3, unless stated otherwise (Figures 1E–G and 6A,C,D,F). T cells were cultured in 96-well round-bottom or 6-well flat-bottom plates (Greiner) and 200 µl or 4 ml RPMI 1640, respectively, supplemented with 10% FCS (v/v), 1 mM sodium pyruvate, 1 mM HEPES, 2 mM GlutaMAX, 100 U/ml penicillin–streptomycin, 0.1 mg/ml gentamicin, 0.1 mM non-essential amino acids, and 0.55 mM β-mercaptoethanol (all Invitrogen). For TCR stimulation, anti-CD3/CD28-coated Dynabeads^®^ (2 beads/cell, Invitrogen) were used at a density of 5 × 10^4^ T cells/well. Where indicated, cultures were supplemented with recombinant human IL-2 (100 U/ml; Teceleukin; Roche), human TGF-β (0.5 or 5 ng/ml; Pepro-Tech), or 5-aza-dC (InvivoGen) at indicated concentrations. For some cultures, neutralizing mAbs to IL-2 (clone S4B6) or TGF-β (clone 1D11.16.8) or a selective TGF-βR inhibitor (SB431542, Sigma-Aldrich) were added at indicated concentrations.

### Gene Expression Analysis

Total populations of viable cells were FACS purified from TCR stimulation cultures at indicated time points, and total RNA was extracted using the RNeasy^®^ Mini Kit and on-column DNase I digestion (QIAGEN). For real-time RT-PCR, cDNA was synthesized using SuperScript II reverse transcriptase (Invitrogen) according to the manufacturer’s recommendations. The QuantiFast SYBR Green PCR kit (Qiagen) and a Mastercycler ep realplex thermal cycler (Eppendorf) were used to analyze cDNA in replicates. The following primers were used: HPRT: 5′-GTC AAC GGG GGA CAT AAA AG-3′ and 5′-AGG GCA TAT CCA ACA ACA AAC-3′; Foxp3: 5′-CCC AGG AAA GAC AGC AAC CTT-3′ and 5′-CAA ACA GGC CGC CGT CTG GAG CC-3′; Smad7: 5′-AAG AGG CTG TGT TGC TGT GA-3′ and 5′-CAG CCT GCA GTT GGT TTG AGA-3′.

### Statistical Analysis

**Figure 2:**
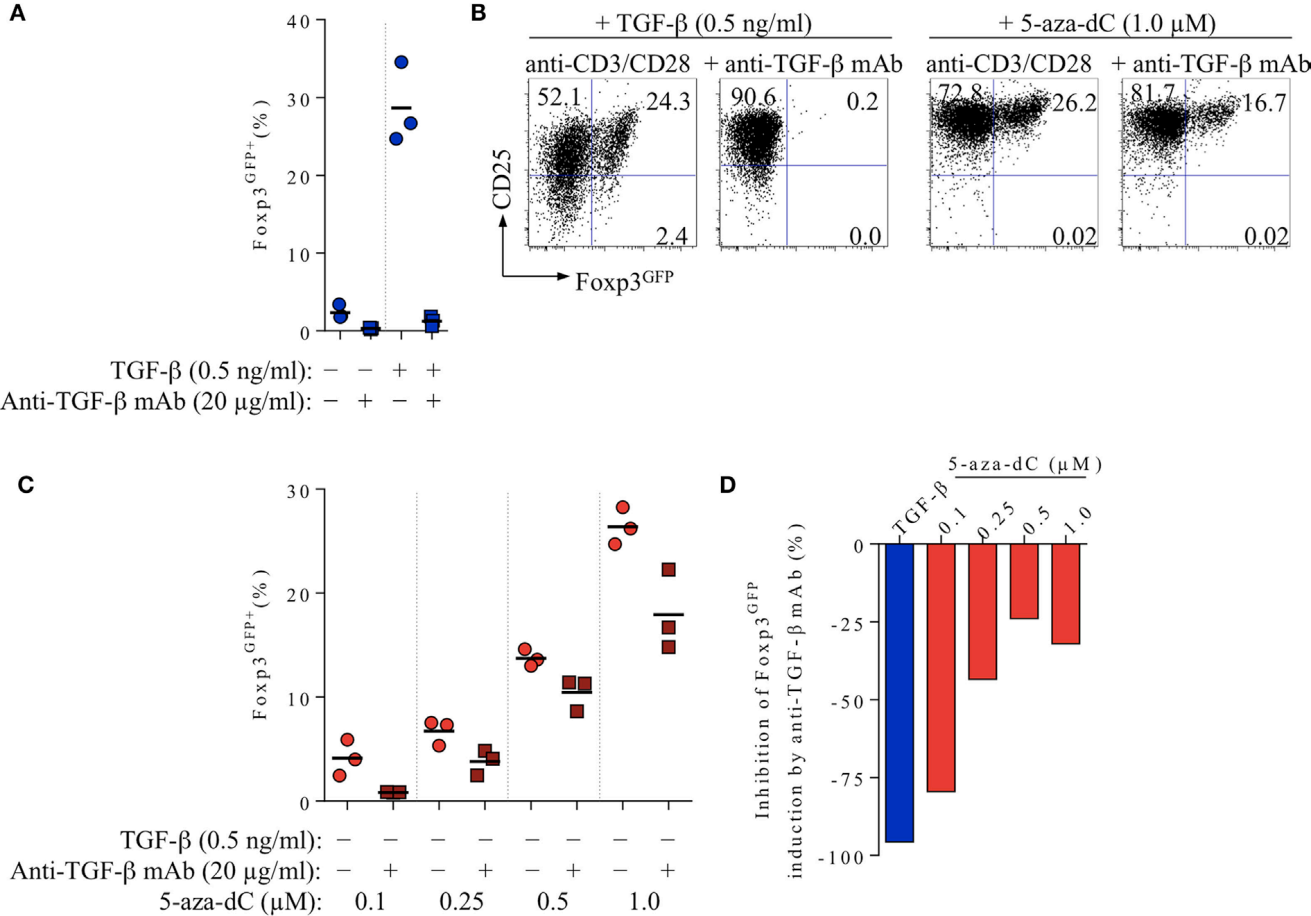
Impact of FCS-/T cell-derived TGF-β on 5-aza-2′-deoxycytidine (5-aza-dC)-mediated Foxp3^+^ iTreg cell generation, in the absence of exogenously added TGF-β. CD4^+^Foxp3^GFP−^ T cell stimulation cultures (for experimental details, see Figure 1) were either left untreated or supplemented with added TGF-β (0.5 ng/ml) or titrating amounts of 5-aza-dC (0.1, 0.25, 0.5, or 1.0 µM), with or without neutralizing anti-TGF-β mAbs (20 µg/ml), as indicated. Cultures were analyzed at day 3 for Foxp3^GFP^ and CD25 expression among gated CD4^+^ T cells. Note that Figure S1 in Supplementary Material depicts experimental details on anti-TGF-β mAb titration. **(A)** 20 µg/ml anti-TGF-β mAb efficiently abrogates Foxp3 induction in T cell receptor stimulation cultures without (left) or with (right) exogenously added TGF-β (0.5 ng/ml). **(B–D)** Inhibition of 5-aza-dC-driven Foxp3^+^ iTreg cell generation by anti-TGF-β mAb-mediated TGF-β blockage, in the absence of added TGF-β. **(B)** Representative flow cytometry of Foxp3^GFP^/CD25 expression among gated CD4^+^ T cells, and **(C)** composite percentages of CD4^+^Foxp3^GFP+^ iTreg cells at indicated culture conditions. **(D)** Percentage inhibition of 5-aza-dC-mediated Foxp3^GFP+^ iTreg cell generation by TGF-β blockage (see Materials and Methods for details). Symbols and horizontal lines **(A,C)** indicate triplicate wells and mean values, respectively. Numbers in dot plots **(B)** indicate the percentages of cells within the respective quadrant. Data are representative of at least two independent experiments.

**Figure 3:**
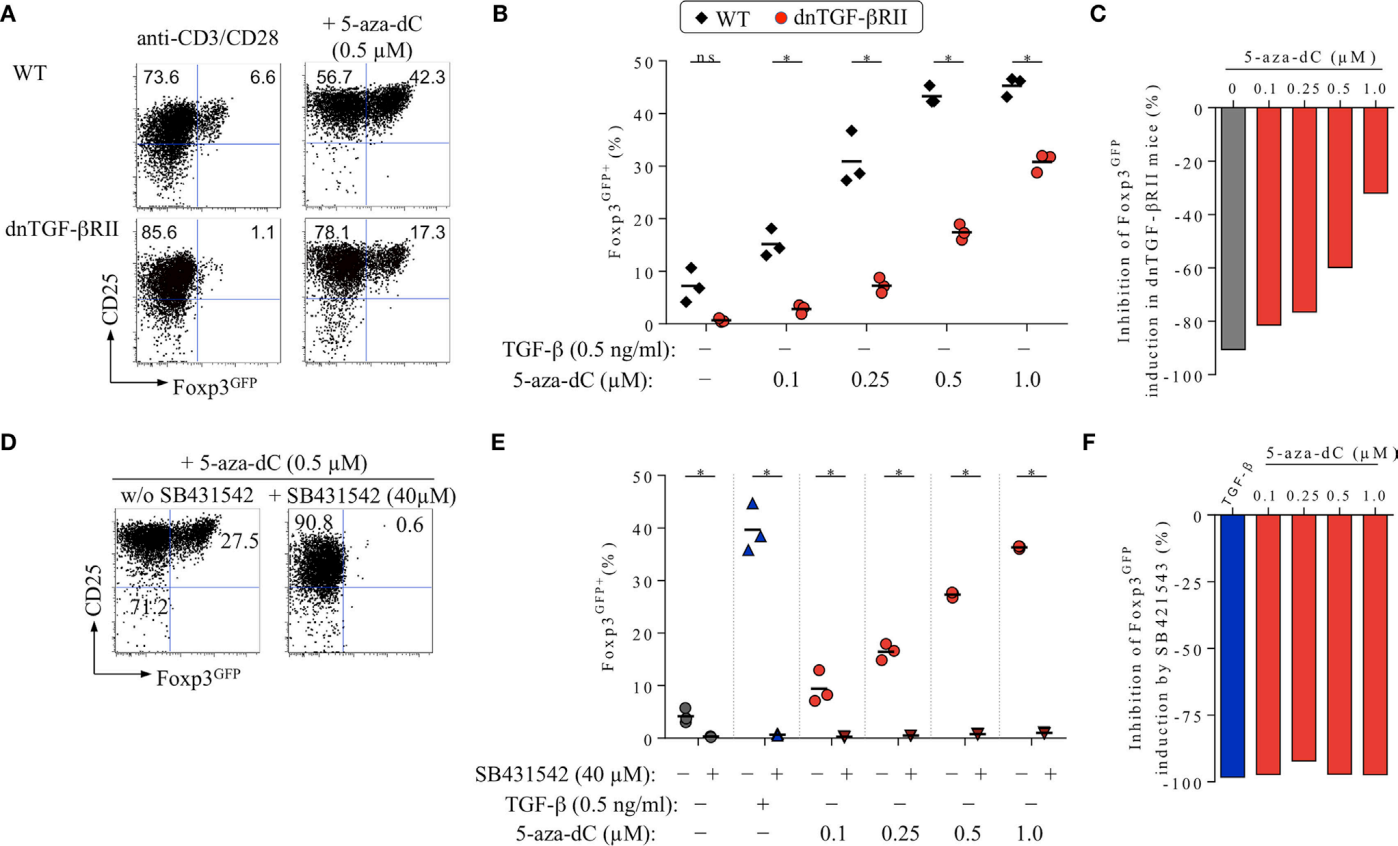
Impact of TGF-βRII signaling on 5-aza-2′-deoxycytidine (5-aza-dC)-mediated Foxp3^+^ iTreg cell generation. See Figure 1 for experimental details on naive CD4^+^Foxp3^GFP−^ T cell isolation. T cell receptor (TCR) stimulation cultures were analyzed on day 3 for Foxp3^GFP^ and CD25 expression among gated CD4^+^ T cells. **(A–C)** Genetic abrogation of TGF-βR signaling. In the absence of added TGF-β, CD4^+^Foxp3^GFP−^ T cells expressing a wild-type (WT) TGF-βRII or a dominant-negative TGF-βRII (dnTGF-βRII) were TCR stimulated in the absence or presence of titrating amounts of 5-aza-dC (0.1, 0.25, 0.5, or 1.0 µM). **(A)** Representative flow cytometry and **(B)** composite percentages of CD4^+^Foxp3^GFP+^ iTreg cells at indicated culture conditions. **(C)** Percentage inhibition of 5-aza-dC-mediated Foxp3^GFP+^ iTreg cell generation by dnTGF-βRII expression (see Materials and Methods for details). **(D–F)** Pharmacological abrogation of TGF-βR signaling. TGF-βR signaling-proficient, naive CD4^+^Foxp3^GFP−^ T cells were subjected to TCR stimulation, either alone or with TGF-β (0.5 ng/ml) or titrating amounts of 5-aza-dC (0.1, 0.25, 0.5, or 1.0 µM), in the absence and presence of 40 µM SB431542, as indicated. Note that Figure S2 in Supplementary Material depicts experimental details on the titration of SB431542. **(D)** Representative flow cytometry and **(E)** composite percentages of CD4^+^Foxp3^GFP+^ iTreg cells at indicated culture conditions. **(F)** Percentage inhibition of 5-aza-dC-mediated Foxp3^GFP+^ iTreg cell generation by SB431542 (see Materials and Methods for details). Numbers in dot plots **(A,D)** indicate the percentages of cells within the respective quadrant. Symbols and horizontal lines **(B,E)** indicate triplicate wells and mean values, respectively. **(B,E)** The level of significance was determined by multiple *t*-tests (unpaired). *P ≤ 0.05. Data are representative of at least two independent experiments.

**Figure 4:**
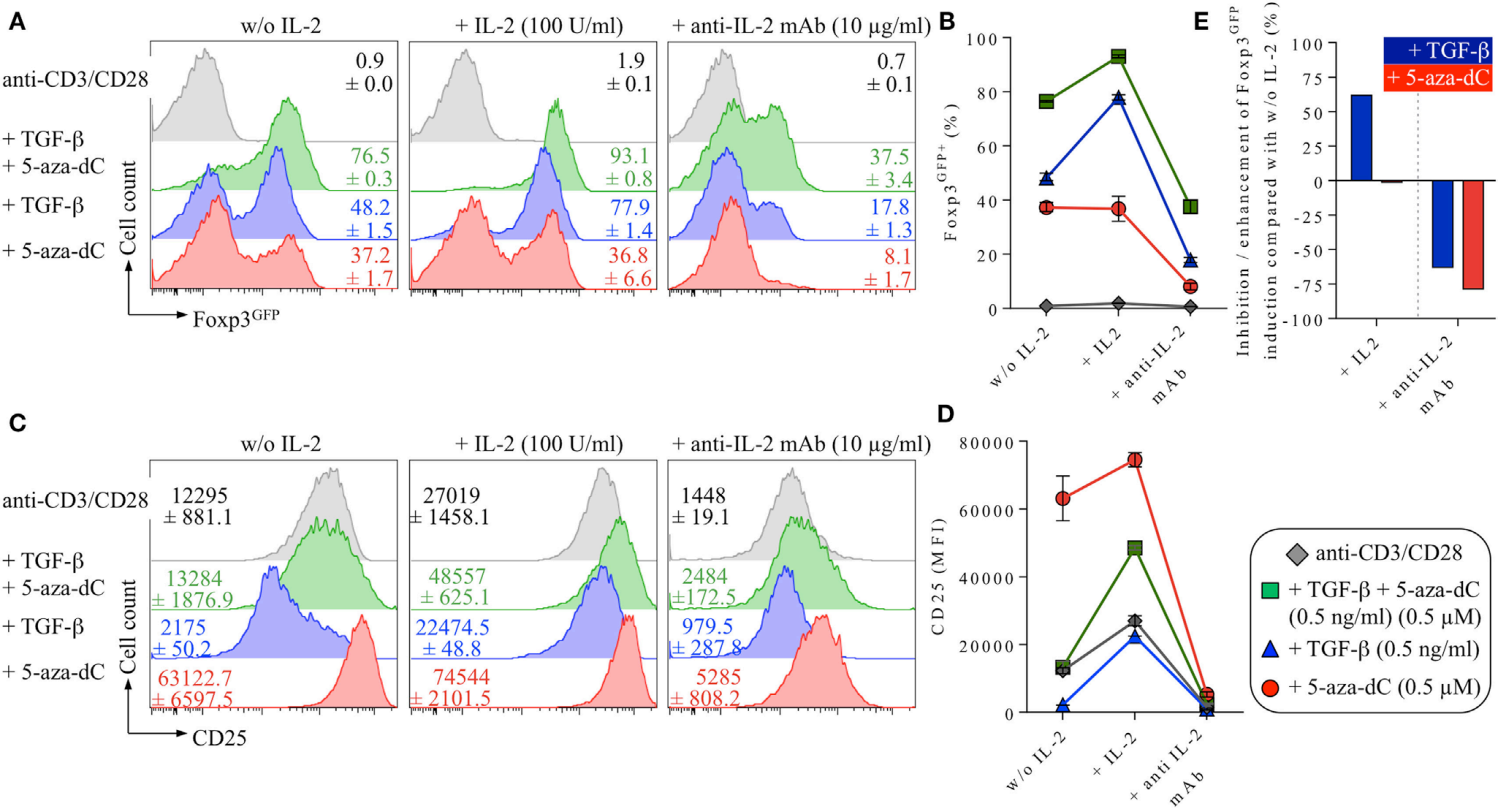
Impact of IL-2 and IL-2R signaling on 5-Aza-2′-deoxycytidine (5-aza-dC)-mediated Foxp3^+^ iTreg cell generation. Naive CD4^+^Foxp3^GFP−^ T cells were T cell receptor stimulated with anti-CD3/CD28-coated beads either alone (shown in gray) or with added TGF-β and 5-aza-dC (0.5 ng/ml and 0.5 µM; shown in green), added TGF-β (0.5 ng/ml; shown in blue) or 5-aza-dC (0.5 µM; shown in red). As indicated, either IL-2 (100 U/ml) or neutralizing anti-IL-2 mAb (10 µg/ml) were added. Cultures were analyzed on day 3 for Foxp3^GFP^ and CD25 expression among gated CD4^+^ T cells. **(A)** Representative histograms of Foxp3^GFP^ expression among gated CD4^+^ T cells and **(B)** composite percentages of CD4^+^Foxp3^+^ iTreg cells at indicated culture conditions. **(C)** Representative histograms and **(D)** median fluorescence intensities (MFIs) of CD25 expression among gated CD4^+^ T cells. **(E)** Percentage inhibition/enhancement of TGF-β-mediated (blue bars) and 5-aza-dC-mediated (red bars) Foxp3^GFP+^ iTreg cell generation by the addition of exogenous IL-2 (left) or anti-IL-2 mAbs (right), as indicated (see Materials and Methods for details). Numbers in histograms show **(A)** the mean values of the percentage of Foxp3^GFP+^ cells and **(C)** MFI values of CD25 expression ±SD at indicated culture conditions. Graphs **(B,D)** depict mean ± SD. Data are representative of two independent experiments.

Statistical significance was assessed using Prism 6 software (GraphPad) and *t*-test (unpaired) with Sidak–Bonferroni corrections for multiple comparisons for determination of statistical significance or two-way ANOVA (ordinary or repeated measures) and Tukey’s multiple comparison test, as indicated. The formula used to calculate percent inhibition of Foxp3^+^ iTreg cell generation was [(*Y* − *X*)/*X*] × 100, where *X* is the proportion CD4^+^ T cells that exhibited Foxp3 expression under culture conditions for efficient Foxp3^+^ iTreg cell generation (i.e., TCR stimulation with either TGF-β or 5-aza-dC and expression of a wild-type TGF-βRII), and *Y* is the proportion CD4^+^ T cells that exhibited Foxp3 expression under inhibitory or enhancing conditions [i.e., TCR stimulation with TGF-β or 5-aza-dC, in the presence of anti-TGF-β mAb (Figure 2D), dnTGF-βRII (Figures 3A–C), SB431542 (Figures 3D–F), anti-IL-2 mAbs, or added IL-2 (Figure 4)].

## Results

### Interplay of 5-Aza-dC with IL-2R/TGF-βR Signaling during Foxp3^+^ iTreg Cell Generation

The generation of Foxp3^+^ iTreg cells from initially naive, TCR-stimulated CD4^+^Foxp3^−^ T cells *in vitro* has been shown to be strictly dependent on IL-2R and TGF-βR signaling (14). In the absence of exogenously added IL-2, production of IL-2 by TCR-stimulated CD4^+^Foxp3^GFP−^ T cells can drive TGF-β-mediated Foxp3^+^ iTreg cell generation in an autocrine manner, but results in a predominantly CD25^low^ phenotype (Figure 1A), indicating limited IL-2 availability at day 3 of such cultures. In contrast, in the absence of added IL-2 and TGF-β, TCR stimulation in the presence of 5-aza-dC promotes the generation of Foxp3^GFP+^ iTreg cells with a CD25^high^ phenotype (Figure 1A). Titration experiments (5-aza-dC: 0.1, 0.25, 0.5, and 1.0 µM) revealed a positive correlation between 5-aza-dC concentrations and proportions of Foxp3^GFP+^ iTreg cells (Figure 1B), whereas cell viability inversely correlated with the amount of added 5-aza-dC (Figures 1C,D). Importantly, the population size of Foxp3^GFP+^ cells was found to be essentially identical, irrespective of whether live/dead cell discrimination was based on FSC/SSC gating alone (Figures 1A–D) or a viability dye (DAPI) was included (data not shown).

Interestingly, the addition of low (0.5 ng/ml; data not shown) or high (5 ng/ml) amounts of TGF-β to 5-aza-dC-supplemented cultures resulted in intermediate expression levels of CD25 on *de novo* generated Foxp3^GFP+^ iTreg cells (Figure 1A), increased proportions of Foxp3^GFP+^ cells over the entire range of tested 5-aza-dC concentrations (Figures 1A,B) (45), and markedly augmented cell viability at high 5-aza-dC concentrations (Figures 1C,D). As an example, the frequency of Foxp3^GFP+^ iTreg cells in TCR stimulation cultures supplemented either with TGF-β only (57.9 ± 6.4%) or with 5-aza-dC only (1.0 µM; 47.8 ± 1.96%) was increased to 73.3 ± 1.1% upon co-administration of both TGF-β and 5-aza-dC (Figure 1B). Hence, 5-aza-dC synergizes with TGF-β to promote the generation of Foxp3^+^CD25^+^ iTreg cells *in vitro*.

Mechanistically, the data presented in Figure 1 suggest that the mode of action of 5-aza-dC may involve the modulation of IL-2R and TGF-βR signaling, two signaling pathways with well-established and non-redundant roles in Foxp3 induction and Foxp3^+^ iTreg cell generation. Our hypothesis that 5-aza-dC may act through indirect mechanisms could be further corroborated by kinetics studies on the expression of *Foxp3* mRNA (quantitative RT-PCR) and Foxp3 protein (Foxp3^GFP^ fluorescence) at early time points during 5-aza-dC- and TGF-β-driven Foxp3^+^ iTreg cell generation (Figures 1E–G). Overall, the results revealed a considerable delay in the kinetics of 5-aza-dC-induced expression of *Foxp3* mRNA (Figure 1E) and Foxp3 protein (Figures 1F,G) although the proportions of Foxp3^GFP+^ iTreg cells were found to be largely comparable at 72 h (5-aza-dC: 35.2 ± 2.1%; TGF-β: 34.7 ± 1.0%). Specifically, while *Foxp3* mRNA expression levels in *ex vivo* CD4^+^Foxp3^GFP−^ T cells (CD25^−^CD62L^high^) were below the level of detection (data not shown), TCR stimulation in the presence of exogenous TGF-β resulted in low but clearly detectable amounts of *Foxp3* mRNA as early as 18 h after initiation of cultures, which further increased over time (Figure 1E; blue triangles) and correlated well with Foxp3^GFP^ protein expression (Figures 1F,G). In contrast, during 5-aza-dC-mediated iTreg cell generation, *Foxp3* mRNA became first detectable at 40 h (Figure 1E; red circles), with expression levels at 48 h remaining 5.6-fold below those observed during TGF-β-mediated iTreg cell generation. Consistently, the kinetics of 5-aza-dC-induced Foxp3^GFP^ protein expression was substantially delayed, with undetectable protein expression levels at 24 and 30 h (Figures 1F,G).

Overall, the delayed kinetics of 5-aza-dC-mediated Foxp3^+^ iTreg cell generation is consistent with a scenario, in which 5-aza-dC promotes Foxp3 expression primarily through indirect mechanisms, such as the enhancement of IL-2R/TGF-βR signaling, rather than acting directly on the *Foxp3* gene locus. Interestingly, quantitative RT-PCR interrogating the expression of genes encoding essential components of the TGF-βR signaling pathway indicated that, in striking contrast to TGF-β-supplemented cultures, 5-aza-dC fails to upregulate of *Smad7* mRNA levels in TCR-stimulated CD4^+^ T cells (Figure 1H). Thus, while TGF-β-mediated iTreg cell induction is associated with Smad7-mediated negative regulation of TGF-βR signaling, the lack of *Smad7* mRNA up-regulation during 5-aza-dC-mediated iTreg cell induction may provide a molecular basis for sustained sensitivity of CD4^+^ T cells to TGF-β during the conversion process in the absence of added TGF-β.

### Impact of TGF-βR Signaling on 5-Aza-dC-Driven Foxp3^+^ iTreg Cell Generation

To further dissect the role of TGF-βR signaling in 5-aza-dC-mediated Foxp3 induction *in vitro*, we performed a series of loss-of-function studies that involved mAb-mediated TGF-β blockage as well as genetic and pharmacological abrogation of TGF-βR signaling.

#### Neutralizing Anti-TGF-β mAbs

In the absence of added TGF-β, TCR stimulation of highly pure populations of truly naive CD4^+^Foxp3^GFP−^ T cells (CD25^−^CD62L^high^) *in vitro* can give rise to low frequencies (≤10%) of newly generated Foxp3^+^ cells (Figure 1A), which can be attributed to the developmental progression of *in vivo* precommitted CD4^+^Foxp3^−^ pTreg cell precursors (1) and/or the presence of basal levels of bioactive TGF-β in bovine serum products (ranging from 2 to 25 ng/ml) used as cell culture supplement (46, 47). In fact, the induction of Foxp3 expression observed in the absence of added TGF-β (2.4 ± 0.9%) was essentially completely abrogated (0.3 ± 0.1%) by high doses of neutralizing anti-TGF-β mAbs (20 µg/ml; clone 1D11.16.8; Figure 2A). Titration experiments (anti-TGF-β mAb: 0.01, 0.1, 1.0, 10.0, or 20.0 µg/ml; Figure S1 in Supplementary Material) further revealed that 20 µg/ml anti-TGF-β mAbs reduces the induction of Foxp3 expression by added TGF-β (28.7 ± 5.2%) to a minimum (1.3 ± 0.6%; Figures 2A,B, left). To assess the relative contribution of FCS/T cell-derived TGF-β to 5-aza-dC-mediated Foxp3 induction (i.e., in the absence of added TGF-β), we then performed TCR stimulation cultures with initially CD4^+^Foxp3^GFP−^ T cells and titrating amounts of 5-aza-dC, in the absence or presence of the neutralizing anti-TGF-β mAb (Figures 2B–D). In these experiments, addition of anti-TGF-β mAbs reduced the efficiency of 5-aza-dC-mediated Foxp3 induction (Figure 2B, right) over the entire range of tested 5-aza-dC concentrations (Figures 2C,D), although to varying degrees: high efficiency (79.5%) of anti-TGF-β mAb-mediated inhibition of Foxp3 induction at low 5-aza-dC concentrations (0.1 µM) could be partially overcome by increasing amounts of 5-aza-dC (e.g., 32.1% inhibition at 1.0 µM 5-aza-dC; Figure 2D). These data indicate that, in the absence of added TGF-β, low levels of FCS-/T cell-derived TGF-β markedly contribute to 5-aza-dC-mediated induction of Foxp3 expression *in vitro*.

#### Genetic Abrogation of TGF-βR Signaling

To directly assess the contribution of TGF-βR signaling to 5-aza-dC-mediated Foxp3^+^ iTreg cell generation, we employed Foxp3^GFP^ mice that additionally expressed a dominant-negative form of the TGF-βRII (dnTGF-βRII) in all CD4^+^ and CD8^+^ T cells (Foxp3^GFP^ × dnTGF-βRII), leading to abrogation of TGF-βR assembly and signal transduction (44). Consistent with our previous experiments (Figures 1 and 2), we isolated through FACS highly pure populations of CD4^+^Foxp3^GFP−^ T cells with a naive phenotype (CD25^−^CD62L^high^) from pooled lymph nodes and spleen of adult Foxp3^GFP^ × dnTGF-βRII mice and performed TCR stimulation cultures, in the absence or presence of titrating amounts of 5-aza-dC (0.1, 0.25, 0.5, and 1.0 µM; Figures 3A–C). Naive CD4^+^Foxp3^GFP−^ T cells from TGF-βR signaling-proficient Foxp3^GFP^ mice were included for comparison. In the absence of 5-aza-dC (Figure 3A, left; Figure 3B), the transgenic dnTGF-βRII completely abrogated basal induction of Foxp3^GFP^ (wild-type: 7.2 ± 3.3%; dnTGF-βRII: 0.7 ± 0.4%), corresponding to 90.6% inhibition (Figure 3C, gray bar). Consistent with mAb-mediated blockade of TGF-β (Figure 2), the transgenic dnTGF-βRII markedly reduced the efficiency of 5-aza-dC-mediated Foxp3 induction (Figure 3A, right; 0.5 µM 5-aza-dC, wild-type: 43.3 ± 1.8%; dnTGF-βRII: 17.4 ± 1.5%) over the entire range of tested 5-aza-dC concentrations (Figure 3B). The dnTGF-βRII-mediated inhibition of 5-aza-dC-driven Foxp3 induction (Figure 3C) was more pronounced at low 5-aza-dC concentrations (0.1 μM: 81.4% inhibition), whereas high amounts of 5-aza-dC partially restored Foxp3^+^ iTreg cell generation (1.0 μM: 32.0% inhibition). These experiments (Figures 3A–C) reveal that, in the absence of added TGF-β, TGF-βR signaling plays a critical role in efficient 5-aza-dC-mediated Foxp3 induction.

Potential reasons for the observed incomplete inhibition of Foxp3 induction in CD4^+^dnTGF-βRII^+^ T cells during 5-aza-dC-driven Foxp3^+^ iTreg cell generation (Figures 3A–C) may include “leakiness” of the dnTGF-βRII approach. In fact, complete abrogation of TGF-βR signaling will critically depend on the stoichiometry of transgenic non-functional dnTGF-βRII and endogenous TGF-βRII protein chains. In light of the inverse relationship between 5-aza-dC concentrations and efficiency of dnTGF-βRII-mediated inhibition of Foxp3 induction (Figures 3B,C), it appears important to note that 5-aza-dC has been reported to increase the amounts of TGF-βRII mRNA and protein (48), but quantitative RT-PCR indicated to us that the addition of 5-aza-dC to TCR-stimulated CD4^+^ T cells has essentially no impact on mRNA expression levels of genes encoding proteins with important functions in TGF-βR signaling (Smad2, Smad3, Smad7, TGF-βR chains, etc.) (Figure 1H, and data not shown). To address the impact of residual TGF-βR signaling activity despite transgenic dnTGF-βRII expression, future studies are warranted employing naive CD4^+^Foxp3^−^ T cells with complete gene-targeted abrogation of endogenous TGF-βRII expression (e.g., by inducible deletion of floxed alleles in mature CD4^+^ T cells by activatable Cre recombinase).

#### Pharmacological Abrogation of TGF-βR Signaling

To ensure complete abrogation of TGF-βRII signaling, we here performed TCR stimulation cultures with TGF-βR signaling-proficient, initially naive CD4^+^Foxp3^GFP−^ T cells and titrating amounts of 5-aza-dC, in the absence or presence of SB431542, a selective and potent inhibitor of TGF-βR activation that interferes with downstream Smad2/3 phosphorylation (Figures 3D–F). Consistent with previous observations (1), our initial titration experiments (Figure S2 in Supplementary Material; SB431542: 0, 2.5, 10, 40, or 80 µM) confirmed that SB431542 inhibits the induction of Foxp3 expression in TCR stimulation cultures with (Figure S2A in Supplementary Material, left) or without added (Figure S2A in Supplementary Material, right) TGF-β in a dose-dependent manner (Figure S2B in Supplementary Material); in TGF-β-supplemented TCR stimulation cultures, SB431542 at a concentration of 40 µM completely abrogated the induction of Foxp3 expression (Figure S2B in Supplementary Material; Figures 3E,F) without compromising overall cell viability (Figure S2C in Supplementary Material). Importantly, in the absence of added TGF-β, 40 µM SB431542 efficiently inhibited 5-aza-dC-mediated Foxp3 induction (Figures 3D,E), even at high concentrations of 5-aza-dC.

Overall, the data presented in Figures 1–3 provide direct evidence of an essential role of TGF-β and TGF-βR signaling in 5-aza-dC-mediated Foxp3^+^ iTreg cell generation.

### Impact of IL-2 and IL-2R Signaling on 5-Aza-dC-Mediated Foxp3^+^ iTreg Cell Generation

#### 5-Aza-dC-Mediated Foxp3^+^ iTreg Cell Generation Is Strictly Dependent on IL-2

Next, we aimed to investigate the role of endogenous IL-2 production (i.e., secreted by TCR-stimulated CD4^+^ T cells) on 5-aza-dC-mediated Foxp3^+^ iTreg cell generation. For this, we compared the impact of enhanced (exogenous IL-2) or abrogated (anti-IL-2 mAbs to block endogenous IL-2) IL-2R signaling on TGF-β- or 5-aza-dC-mediated conversion of naive CD4^+^Foxp3^GFP−^ T cells into Foxp3^GFP+^CD25^+^ iTreg cells (Figure 4). As expected (14, 49), TGF-β-mediated Foxp3^+^ iTreg cell generation (Figure 4A, blue histograms; Figure 4B) was substantially enhanced by the addition of exogenous IL-2 and efficiently abrogated by neutralizing anti-IL-2 mAbs, as judged by the frequency of Foxp3^GFP+^ cells (Figures 4A,B; without IL-2: 48.2 ± 1.5%; + IL-2: 77.9 ± 1.4%; + anti-IL-2 mAb: 17.8 ± 1.3%) and CD25 expression levels (median fluorescence intensity; Figures 4C,D). In contrast, 5-aza-dC-mediated Foxp3^+^ iTreg cell generation (Figure 4A, red histograms; Figure 4B) remained largely unaffected by the addition of exogenous IL-2; importantly, CD25 expression on Foxp3^+^ iTreg cells induced by 5-aza-dC appeared to be uncoupled from the availability of IL-2 (Figures 4C,D): in the absence of added IL-2, CD25 was already expressed at high levels on essentially all cultured CD4^+^ T cells (i.e., irrespective of their Foxp3 expression status) and only marginally increased in IL-2-supplemented cultures despite the addition of excessive amounts of IL-2 (100 U/ml; Figures 4C,D). Nevertheless, in the absence of added IL-2, anti-IL-2 mAb-mediated blockage of endogenously produced IL-2 severely abrogated 5-aza-dC-mediated Foxp3^+^ iTreg cell generation (Figures 4A,B,E). Interestingly, generation of Foxp3^+^ iTreg cells by the synergistic activity of both TGF-β and 5-aza-dC (Figures 4A,B; green histograms/squares) could be further enhanced by added IL-2, but was partially refractory to anti-IL-2-mediated abrogation observed in “TGF-β only” and “5-aza-dC only” cultures.

#### 5-Aza-dC-Mediated Foxp3 Induction in TCR- and IL-2R-Stimulated CD8^+^ T Cells

**Figure 5:**
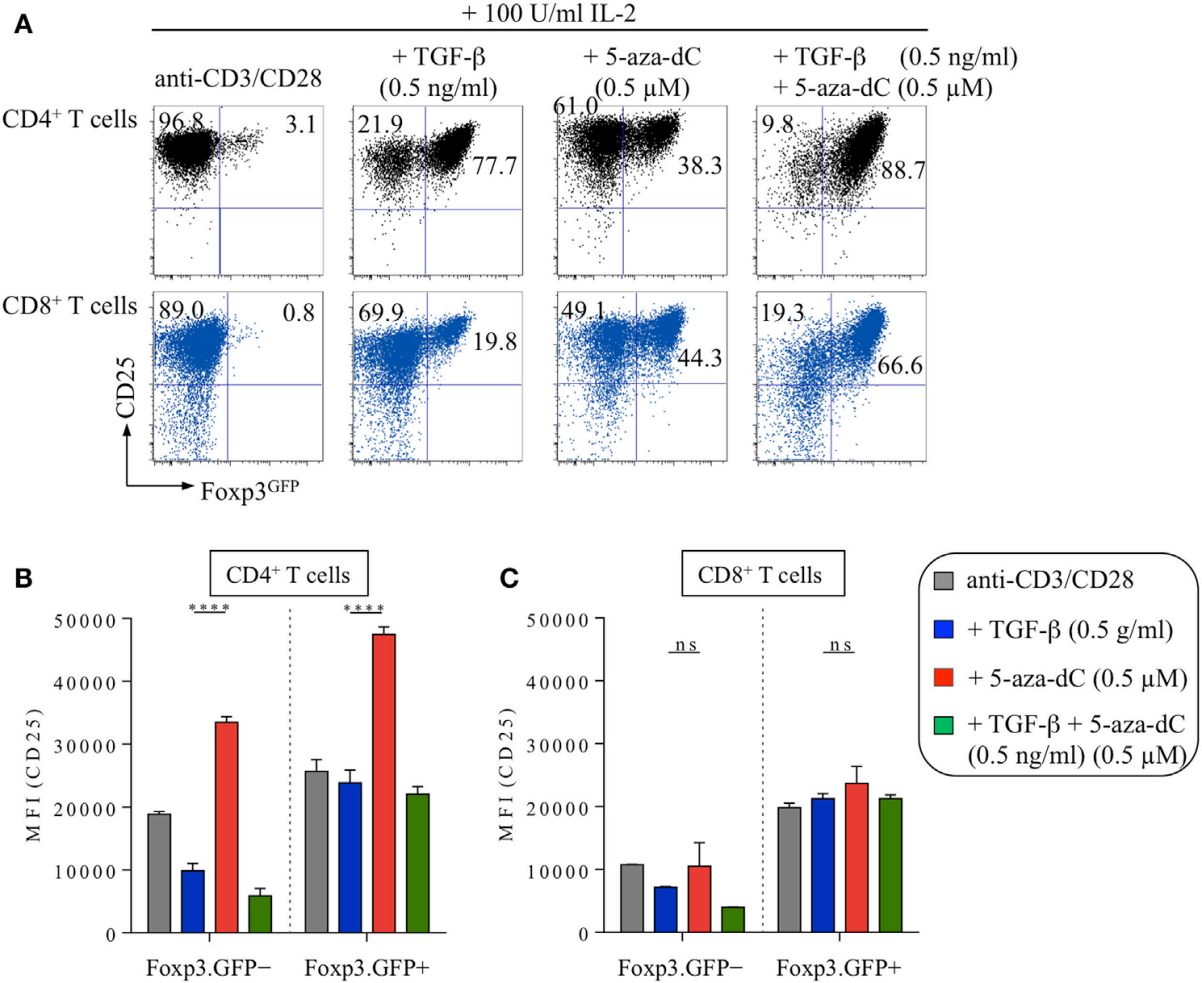
5-Aza-2′-deoxycytidine (5-aza-dC)-mediated induction of Foxp3 expression in CD8^+^ T cells *in vitro*. Naive CD4^+^ and CD8^+^ T cells (both Foxp3^GFP−^CD25^−^CD62L^high^) were FACS-isolated to high purity from pooled lymph nodes and spleen of adult Foxp3^GFP^ mice and subjected to T cell receptor stimulation *in vitro* (anti-CD3/CD28-coated beads) in the presence of IL-2 (100 U/ml), either alone or with added TGF-β (0.5 ng/ml) and/or 5-aza-dC (0.5 µM), as indicated. Cultures were analyzed at day 3 for Foxp3^GFP^ and CD25 expression among gated CD4^+^ or CD8^+^ T cells. **(A)** Representative flow cytometry of Foxp3^GFP^/CD25 expression among gated CD4^+^ (top, black) and CD8^+^ (bottom, blue) T cells. Numbers in dot plots indicate the percentages of cells within the respective quadrant. **(B,C)** Median fluorescence intensities (MFIs) of CD25 expression among gated **(B)** CD4^+^ T cells (left: Foxp3^GFP−^, right: Foxp3^GFP+^) and **(C)** CD8^+^ T cells (left: Foxp3^GFP−^, right: Foxp3^GFP+^). Graphs show mean percentages ±SD of CD25 MFI from triplicate wells. The level of significance was determined by two-way ANOVA with Tukey’s multiple comparison test: ****P ≤ 0.0001 and *P* > 0.05 (ns).

5-Aza-2′-deoxycytidine has been shown to promote Foxp3 expression and CNS2 hypomethylation in a murine CD8^+^ cytotoxic T cell line that normally lacks Foxp3 expression and exhibits a highly methylated CNS2 (41). The essential role of Dnmt activity and CpG methylation in repressing Foxp3 expression was further corroborated by the observation that genetic ablation of Dnmt1 facilitates the upregulation of Foxp3 expression in initially naive Foxp3^−^ T cells of both the CD4 and CD8 lineage *in vitro*, on TCR and IL-2R stimulation in the absence of added TGF-β (40). Here, we show that these observations can be recapitulated by pharmacological inhibition of CpG methylation in freshly isolated CD8^+^ T cells (Figure 5A; top: CD4^+^ T cells, bottom: CD8^+^ T cells). To enable a direct comparison of 5-aza-dC-driven Foxp3 induction in initially naive, TCR-stimulated CD4^+^ and CD8^+^ T cells, all cultures were supplemented with exogenous IL-2 (100 U/ml), as IL-2 represents an essential survival/growth factor for CD8^+^ T cells and highly pure populations of naive CD8^+^ T cells fail to produce adequate amounts of IL-2 under given TCR stimulatory conditions. In IL-2-supplemented CD8^+^ T cell stimulation cultures (Figure 5A, bottom), the frequencies of TGF-β-induced Foxp3^+^ T cells (24.9 ± 0.2%) were almost doubled by 5-aza-dC (42.5 ± 17.1%) and increased to 75.3 ± 2.9% upon co-administration of TGF-β and 5-aza-dC (Figure 5A), demonstrating that 5-aza-dC-mediated inhibition of CpG methylation is suitable to promote Foxp3 expression in both CD4^+^ and CD8^+^ T cells. However, we consistently observed significantly increased expression levels of CD25 upon 5-aza-dC-mediated Foxp3 induction in CD4^+^ T cells (Figure 5B) but not in CD8^+^ T cells (Figure 5C), suggesting that 5-aza-dC is acting on IL-2 expression and/or IL-2R signaling, rather than directly acting on *Cd25* gene expression. Furthermore, we did not observe any evidence for Foxp3 induction in LPS-stimulated CD19^+^ B cells in the presence of TGF-β and/or 5-aza-dC (data not shown), indicating that 5-aza-dC-mediated Foxp3 induction is restricted to the T cell lineage.

#### 5-Aza-dC Enhances the Expression of CD25 and pSTAT5

**Figure 6:**
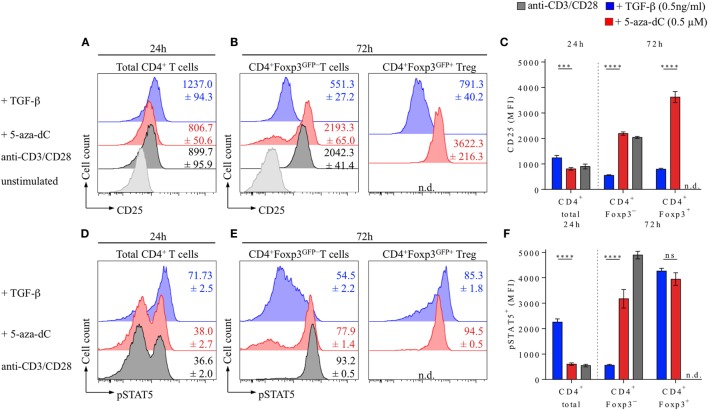
5-Aza-2′-deoxycytidine (5-aza-dC) promotes IL-2R signaling during Foxp3^+^ iTreg cell generation by enhancing CD25 and pSTAT5 expression. Naive CD4^+^Foxp3^GFP−^ T cells were T cell receptor stimulated (see Figure 1 for experimental details) either alone or in the presence of TGF-β (0.5 ng/ml) or 5-aza-dC (0.5 µM). **(A–C)** Kinetics of CD25 expression. **(A)** Representative flow cytometry of CD25 expression among gated total CD4^+^ T cells at 24 h, **(B)** CD4^+^Foxp3^GFP−^ and CD4^+^Foxp3^GFP+^ T cells at 72 h after initiation of cultures, and **(C)** corresponding median fluorescence intensities (MFIs) of CD25 expression (left: 24 h, CD4^+^ total; right: 72 h, CD4^+^Foxp3^GFP−^ and CD4^+^Foxp3^GFP+^). **(D–F)** STAT5 phosphorylation. **(D)** Representative flow cytometry of pSTAT5 expression among gated total CD4^+^ T cells at 24 h and **(E)** CD4^+^Foxp3^GFP−^ and CD4^+^Foxp3^GFP+^ T cells at 72 h after initiation of cultures, and **(C)** corresponding MFIs of pSTAT5 expression (left: 24 h, CD4^+^ total; right: 72 h, CD4^+^Foxp3^GFP−^ and CD4^+^Foxp3^GFP+^). Numbers in histograms and graphs show mean percentages ± SD of **(A–C)** CD25 and **(D–F)** pSTAT5 MFIs from triplicate wells [not detectable (n.d.)]. **(C,F)** The level of significance was determined by two-way ANOVA with Tukey’s multiple comparison test: ****P ≤ 0.0001, ***P ≤ 0.001, and *P* > 0.05 (ns). Data are representative of two independent experiments.

The expression of CD25 on T cells is regulated by multiple mechanisms, including TCR signal strength, Foxp3 occupancy of regulatory regions within the *Cd25* gene, and IL-2R signaling, with IL-2 providing a positive feedback loop that involves STAT5 binding to the *Cd25* gene locus (50). Therefore, we next sought to correlate the expression of Foxp3, CD25, and pSTAT5 at early (24 h) and late (72 h) time points during TGF-β- and 5-aza-dC-mediated Foxp3^+^ iTreg cell generation in the absence of added IL-2, i.e., experimental conditions under which IL-2 availability is limited (Figure 6). 24 h after initiation of naive CD4^+^Foxp3^GFP−^ T cell stimulation cultures, expression of Foxp3^GFP^ protein remained largely undetectable (<3.0%, data not shown), but essentially all CD4^+^ T cells already upregulated CD25 expression (Figures 6A–C), irrespective of whether TCR stimulation cultures were left untreated (gray histograms) or supplemented with either TGF-β (blue histograms) or 5-aza-dC (red histograms); at this early time point, the majority of CD4^+^ T cells that had been TCR stimulated in the presence of TGF-β exhibited high expression levels of pSTAT5 (Figures 6D,F), while the pSTAT5 expression pattern of TCR-/5-aza-dC-stimulated T cells closely resembled that of untreated T cell stimulation cultures. After 72 h, and consistent with Foxp3 promoter occupancy stabilizing and amplifying *Cd25* gene expression (51), TGF-β-induced Foxp3^GFP^ expression correlated with high expression levels of CD25 (Figure 6B, blue histograms) and pSTAT5 (Figure 6E, blue histograms), while CD4^+^Foxp3^GFP−^ T cells exhibited somewhat lower CD25 expression levels and only limited STAT5 phosphorylation; in striking contrast, TCR stimulation in the presence of 5-aza-dC promoted high expression levels of CD25 (Figure 6B, red histograms) and pSTAT5 (Figure 6E, red histograms) in CD4^+^Foxp3^GFP−^ T cells that was further increased in CD4^+^Foxp3^GFP+^ T cells. Thus, under experimental conditions of limited IL-2 availability, 5-aza-dC promotes Foxp3^+^ iTreg cell generation by the enhancement of IL-2R signaling.

## Discussion

Here, we provide evidence that in the absence of added TGF-β and IL-2, initiation of Foxp3 expression during 5-aza-dC-mediated Foxp3^+^ iTreg cell generation from TCR-stimulated, initially naive CD4^+^Foxp3^−^ T cells critically depends on TGF-βR and IL-2R signaling, two signaling pathways with well-established roles in Foxp3^+^ iTreg cell generation *in vitro* and pTreg cell generation *in vivo*. Our data are consistent with the interpretation that 5-aza-dC initially facilitates stochastic Foxp3 induction by the enhancement of TGF-βR and IL-2R signaling under experimental conditions of limited TGF-β and IL-2 availability, elucidating an additional mode of action of 5-aza-dC beyond the direct modulation of CNS2 CpG methylation within the *Foxp3* gene locus. Concomitant 5-aza-dC-mediated CNS2 hypomethylation may further stabilize induced Foxp3 expression by transcription factor recruitment, resulting in the accumulation of iTreg cells with a stable Foxp3^+^ phenotype. Considering the dose-dependent cytotoxicity of 5-aza-dC (Figures 1C,D), the relatively high proportions of Foxp3^+^ iTreg cells at later time points of TCR stimulation cultures are likely to reflect a selective survival advantage of cells that succeeded in acquiring a CD25^high^Foxp3^high^ phenotype.

Consistent with this interpretation, several previous observations argue against a scenario, in which pharmacological Dnmt inhibition bypasses essential signaling pathways implicated in the process of extrathymic Foxp3 induction and Foxp3^+^ iTreg cell generation by directly modulating the methylation status of *Foxp3* CpG elements. First, studies using CD4^+^ T cells from gene-targeted mice established a non-redundant role of CNS1 in extrathymic Foxp3 induction (5, 6, 29, 30), whereas the CpG-rich CNS2, which represents the target of 5-aza-dC-mediated hypomethylation, was found to be dispensable in this process (20, 32, 33). Second, TCR-stimulated CD4^+^ effector/memory cells are largely refractory to TGF-β-/IL-2-mediated induction of Foxp3 expression, and 5-aza-dC fails to abrogate this resistance (21), indicating that other signaling pathways apart from CpG demethylation play an essential role in 5-aza-dC-mediated Foxp3 induction. Third, in addition to CD4^+^ T cells, 5-aza-dC can drive aberrant Foxp3 expression in non-CD4^+^ T cells, such as NK cells (52) and CD8^+^ T cells (Figure 5), but rather than indiscriminately activating many loci, 5-aza-dC appears selective in that it affects *Foxp3* gene expression and a limited set of Treg cell signature proteins (such as CD25 and GITR) (21, 53). Notably, on TCR stimulation in the presence of 5-aza-dC, *Foxp3* mRNA expression and CNS2 demethylation were observed only in the fraction of CD4^+^ T cells that expressed Foxp3 protein (20), arguing against overall dysregulated gene expression by 5-aza-dC. Thus, it appears that the impact of 5-aza-dC on DNA methylation is unexpectedly selective, which could at least in part be attributed to the inverse relationship between 5-aza-dC-mediated Foxp3 induction and cell death (20) (Figure 1), which may result in the loss of cells with severely dysregulated gene expression and preferential survival of Foxp3^+^ iTreg cells that maintained largely unperturbed gene expression. Consistently, murine Foxp3^+^ iTreg cells that had been generated by TCR stimulation in the presence of TGF-β in combination with 5-aza-dC have been shown to exhibit potent suppressor function both *in vitro* and upon adoptive transfer *in vivo* (21).

Previous studies employing mice with constitutive *Foxp3*-CNS2 deficiency (Foxp3Δ^CNS2^) highlighted the important role of CNS2 in maintaining Foxp3 expression and thus Treg cell identity *in vivo* and provided direct evidence that efficient 5-aza-dC-mediated Foxp3^+^ iTreg cell generation *in vitro* requires CNS2 (33): under suboptimal iTreg cell differentiation conditions (i.e., addition of 5-aza-dC after 1 day of TCR stimulation for the duration of 24 h, rather than addition of 5-aza-dC at the start until the end of TCR stimulation cultures), up to 15% of CNS2-proficient CD4^+^ T cells with an initially naive Foxp3^GFP−^CD62L^high^CD44^low^ phenotype acquired Foxp3^GFP^ expression, whereas 5-aza-dC-mediated Foxp3^GFP^ induction was completely abrogated in Foxp3Δ^CNS2^ CD4^+^ T cells. Thus, it appears that 5-aza-dC-mediated Foxp3 induction and Foxp3^+^ iTreg cell generation are initiated by low amounts of FCS-/T cell-derived TGF-β/IL-2 and enhanced TGF-βR/IL-2R signaling, while developmental progression is critically dependent on the recruitment of key transcription factors to CNS2. In fact, enforced 5-aza-dC-mediated CNS2 demethylation in viable CD4^+^ T cells was found to be sufficient for CNS2 binding of CREB/ATF (41) and Ets-1 (54). Clearly, future studies are warranted to determine the exact role of CNS2 in 5-aza-dC-mediated Foxp3^+^ iTreg cell generation.

While 5-aza-dC may facilitate TGF-βR signaling in response to limited amounts of serum-derived TGF-β by enhancing TGF-βR expression (both mRNA and protein (48)), the observed increased expression levels of CD25 (Figures 4C,D) and pSTAT5 (Figures 6D,E) by converting CD4^+^ T cells provide a molecular basis for enhanced IL-2R signaling during 5-aza-dC-mediated Foxp3^+^ iTreg cell generation. Considering that TCR-stimulated CD8^+^ T cells fail to secrete significant amounts of IL-2 and lack increased CD25 expression levels in the presence of 5-aza-dC (Figure 5), it appears likely that the enhanced CD25 expression on converting CD4^+^ T cells (Figure 4) is driven by bystander IL-2 production during 5-aza-dC-mediated Foxp3^+^ iTreg cell generation. Consistently, the fraction of CD25^dim^ but not CD25^high^ cells has recently been reported to express significant amounts of IL-2 during 5-aza-dC-mediated iTreg cell generation from human TCR-stimulated CD4^+^CD25^−^ T cells (53). However, currently, we cannot formally exclude that 5-aza-dC may additionally promote CD25 expression through the establishment of a Treg cell-specific CpG hypomethylation pattern within the *Cd25* gene locus (22). Thus, rather than promoting STAT5 phosphorylation through direct mechanisms, 5-aza-dC may indirectly enhance IL-2R signaling by increasing IL-2 production in TCR-stimulated bystander cells and by facilitating sustained CD25^high^ expression on converting cells by demethylation of CpG regions within the *Cd25* gene.

TGF-β-supplemented cultures represent a remarkably robust approach to promote Foxp3 expression in TCR-stimulated, initially naive CD4^+^Foxp3^−^ T cells, but the unstable Foxp3^+^ phenotype of such iTreg cells has represented a major obstacle to clinical translation. In this context, it appears noteworthy that 5-aza-C has been approved by the FDA an epigenetic therapeutic agent to treat some human cancers (39, 55–57). Systemic administration of 5-aza-C has been reported to ameliorate clinical symptoms in murine models of undesired immunity [ovalbumin-induced airway hyperresponsiveness (58) and virus-induced ocular infection (45)], perhaps by promoting the accumulation of Foxp3^+^ Treg cells with increased expression of Treg cell signature proteins (CD25, FR4, and GITR) and suppressor function (45). However, the substantial cytotoxic effects inherent to nucleoside Dnmt inhibitors, which may contribute to their antitumor activity, poses a considerable limitation for their further use as therapeutic agent for *in vivo* administration in clinical settings of unwanted immunity (39).

However, initial reports on 5-aza-C-mediated Foxp3 induction in human CD4^+^ T cells (21, 59) have recently been substantiated by detailed studies (53), indicating that TCR stimulation (in the absence of added TGF-β or IL-2) of initially CD4^+^CD25^−^ T cells in 5-aza-C-supplemented cultures promotes the acquisition of a FOXP3^high^ phenotype with enhanced expression of Foxp3^+^ Treg cell signature proteins (such as CD25 and GITR). Such 5-aza-C-induced human FOXP3^+^ iTreg cells were hyporesponsive to TCR engagement and lacked IL-2 production, but retained Foxp3 expression and suppressor function after IL-2-driven proliferative expansion (53). Thus, the beneficial effect of 5-aza-C on extrathymic Foxp3 induction and its Foxp3-stabilizing ability to promote efficient CNS2 demethylation clearly deserves further consideration as an approach toward the *in vitro* generation of functionally stable Foxp3^+^ iTreg cells for adoptive cell therapy.

## Author Contributions

KF, NL, SD, AIG, and SS designed, performed, and analyzed the experiments. KF and NL contributed to the data interpretation and assisted in manuscript preparation. SS and KK conceived the research, guided its design, analysis and interpretation, and wrote the manuscript.

## Conflict of Interest Statement

The authors declare that the research was conducted in the absence of any commercial or financial relationships that could be construed as a potential conflict of interest. The reviewer MK and handling editor declared their shared affiliation.

## Supplementary Material

Figure S1 Titration of neutralizing anti-TGF-β mAbs to block TGF-β-mediated Foxp3 induction. Naive CD4^+^Foxp3^GFP−^ T cells were T cell receptor (TCR) stimulated in the absence or presence of exogenously added TGF-β and titrating amounts of anti-TGF-β mAbs (clone 1D11.16.8; 0.01, 0.1, 1.0, 10, and 20 µg/ml), as indicated. TCR stimulation cultures were analyzed at day 3 for Foxp3^GFP^ and CD25 expression among gated CD4^+^ T cells. **(A)** Representative flow cytometry (left: 0.5 ng/ml TGF-β; right: without TGF-β) and **(B)** composite percentages of Foxp3^GFP+^ iTreg cell generation at indicated culture conditions. **(C)** Percentage inhibition of 5-aza-dC-mediated Foxp3^GFP+^ iTreg cell generation by TGF-β blockage (see Materials and Methods for details). Numbers in dot plots **(A)** indicate the percentages of cells within the respective quadrant. Symbols and horizontal lines **(B)** indicate triplicate wells and mean values, respectively.

Figure S2 Titration of SB431542 to inhibit TGF-βR signaling during Foxp3^+^ iTreg cell generation. As indicated, naive CD4^+^Foxp3^GFP−^ T cells were T cell receptor stimulated in the presence (+TGF-β; 0.5 ng/ml) or absence (without TGF-β) of exogenously added TGF-β, with or without titrating amounts of SB431542 (2.5, 10, 40, or 80 µM), a selective inhibitor of TGF-βR activation and Smad2/3 phosphorylation. Cultures were analyzed at day 3 for Foxp3^GFP^ and CD25 expression among gated CD4^+^ T cells. **(A)** Representative flow cytometry and **(B)** composite percentages of Foxp3^GFP+^ iTreg cell generation at indicated culture conditions. **(C)** Corresponding composite percentages of viable cells (FSC/SSC). Numbers in dot plots **(A)** indicate the percentages of cells within the respective quadrant. Symbols and horizontal lines **(B,C)** indicate triplicate wells and mean values, respectively.
