# Supplementary figures and images for: Critical Role of TGF-β and IL-2 Receptor Signaling in Foxp3 Induction by an Inhibitor of DNA Methylation
Source: Front Immunol. 2018 Feb 2;9:125. doi: 10.3389/fimmu.2018.00125 (PMC5801288; doi:10.3389/fimmu.2018.00125)

Supplementary Figure 1

A

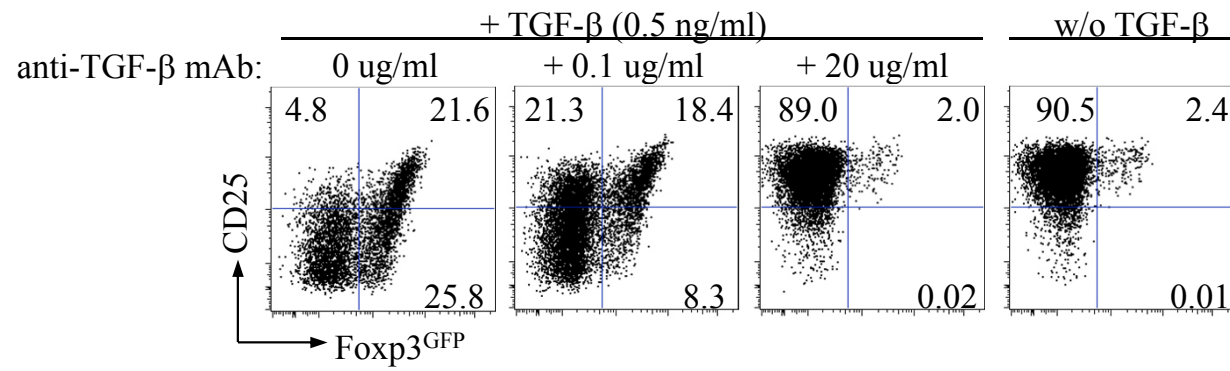

# B

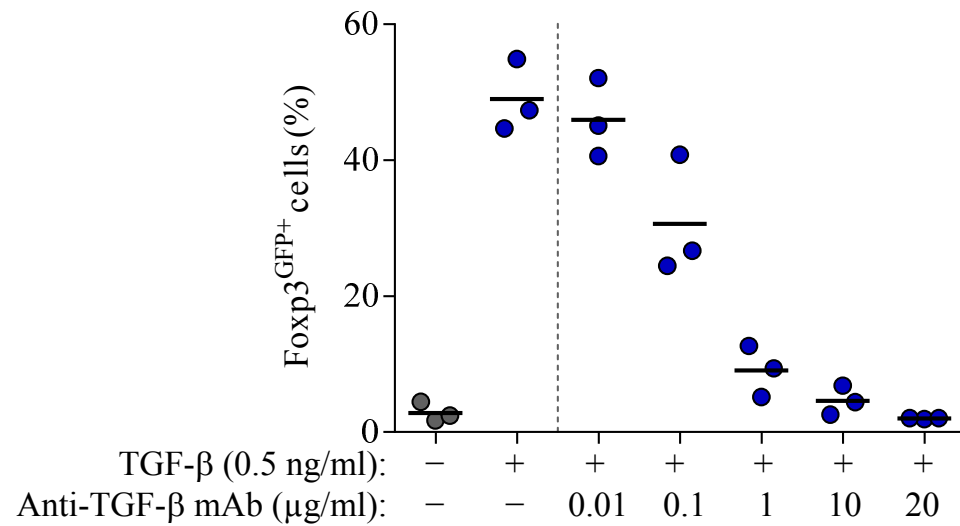

C

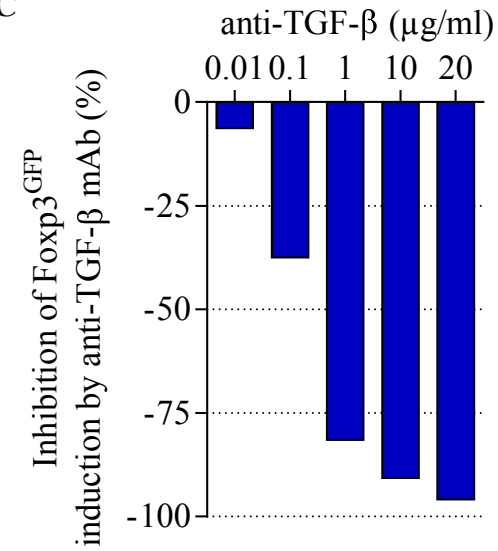

Supplementary Figure 2

A

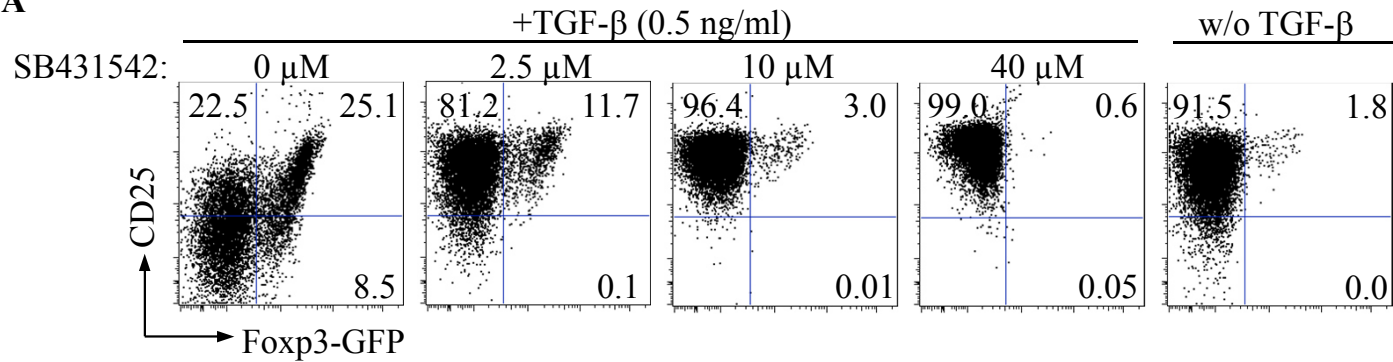

B

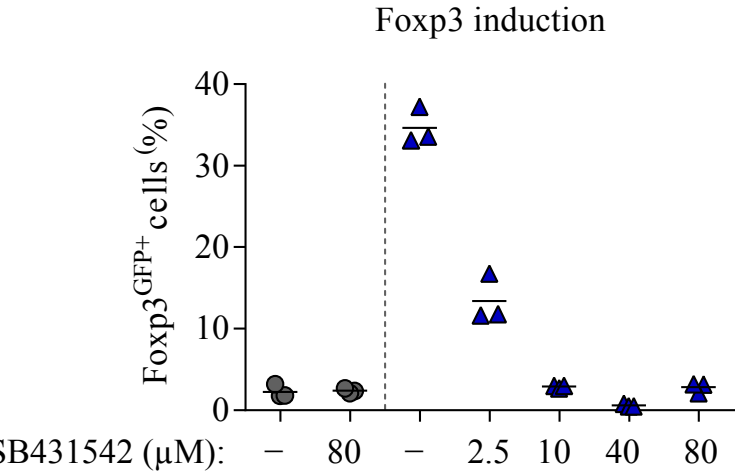

C

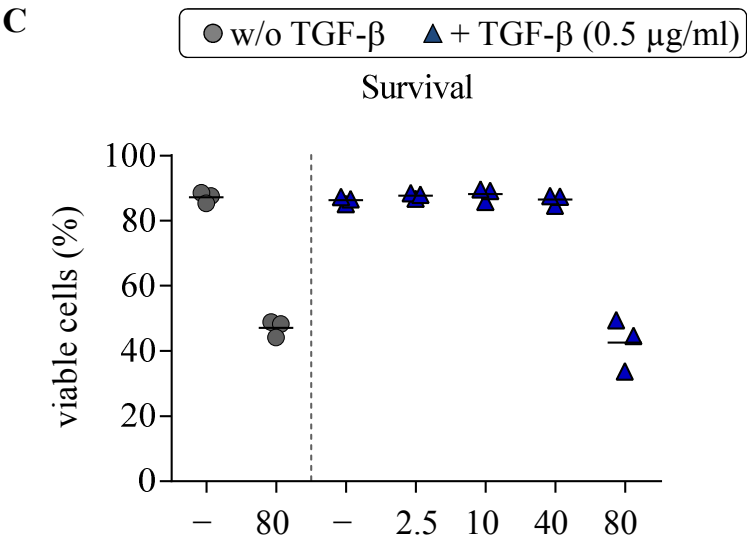

Supplement: Figure S1 — Titration of neutralizing anti-TGF-β mAbs to block TGF-β-mediated Foxp3 induction. Naive CD4+Foxp3GFP− T cells were T cell receptor (TCR) stimulated in the absence or presence of exogenously added TGF-β and titrating amounts of anti-TGF-β mAbs (clone 1D11.16.8; 0.01, 0.1, 1.0, 10, and 20 µg/ml), as indicated. TCR stimulation cultures were analyzed at day 3 for Foxp3GFP and CD25 expression among gated CD4+ T cells. (A) Representative flow cytometry (left: 0.5 ng/ml TGF-β; right: without TGF-β) and (B) composite percentages of Foxp3GFP+ iTreg cell generation at indicated culture conditions. (C) Percentage inhibition of 5-aza-dC-mediated Foxp3GFP+ iTreg cell generation by TGF-β blockage (see Materials and Methods for details). Numbers in dot plots (A) indicate the percentages of cells within the respective quadrant. Symbols and horizontal lines (B) indicate triplicate wells and mean values, respectively. [file Presentation_1.PDF]
